# Deconstructing and Historicizing Access to Medicines: The Changing Priority of Pharmaceutical Governance in China

**DOI:** 10.3389/fsoc.2020.537919

**Published:** 2021-01-29

**Authors:** Lantian Li

**Affiliations:** Northwestern University, Evanston, IL, United States

**Keywords:** access to medicines, pharmaceutical governance, drug availability, drug affordability, China

## Abstract

Securing access to medicines (ATM) is critical for improving public health outcomes. Existing research has long identified and analyzed various barriers that may impede ATM at the global, national, or local levels. However, it tends to adopt a normative perspective to prescribe what infrastructures, resources, and measures *should* be put in place to improve ATM. Little scholarship has explored how and why countries may prioritize certain dimensions of ATM over others in pharmaceutical governance within specific historical contexts. This article fills that gap by deconstructing and historicizing the concept of ATM. The author aims to make two arguments. First, tensions easily arise between different dimensions of ATM, and prioritizing certain dimensions in pharmaceutical policy may impede improvements in others (e.g., availability vs. affordability). Second, which dimension(s) of ATM might be prioritized in the state’s pharmaceutical policy hinges upon social, economic, and political forces. To substantiate these arguments, the author draws on interview and archival evidence from China. Specifically, the author provides a historical account of how and why the priorities of pharmaceutical governance in China changed over time: 1) 1949—late 1970s: pursuing both drug availability and affordability through socialist planning; 2) early 1980s—2015: priority shifting from availability (before the mid-1990s) to affordability (after the mid-1990s); 3) 2015—present: striving for a rebalance between drug availability and affordability.

## Introduction

The entire world has been waiting for the birth of effective covid-19 drug treatments. But normally, a new drug cannot be successfully developed until after years of experimentation, from preclinical molecule screening and animal testing to phase I, II, and III clinical trials. Let us assume one of the current covid-19 drug candidates turns out to be the perfect cure. Will this scientific breakthrough help improve access to medicines (ATM) for people all over the world? It largely depends on which country one resides in. First, the drug must obtain marketing approval from a certain country’s regulatory agency. The application must provide substantial and convincing clinical data in compliance with specific registration guidelines. Second, the drug must be manufactured on a large scale and distributed widely across the country; otherwise, it would be inaccessible to people in remote and/or underdeveloped areas. Third, adequate protocols and regulations must be enforced to guarantee appropriate levels of drug safety, quality, efficacy, and rational use. Last, the price of the drug and out-of-pocket payments must be set within a reasonable range so that even households living in poverty can afford it.

Ideally, only after satisfying all of these requirements can a country claim to have achieved a significant improvement in ATM—in this case, access to covid-19 drug treatment—for the majority of its citizens. These requirements, in my view, correspond to the four dimensions of ATM: 1) availability: the extent to which an innovative drug can be developed and produced in a timely manner; 2) accessibility: the extent to which a drug can be widely distributed and delivered within a territory; 3) appropriateness: the extent to which a drug is safe, efficacious, qualified, and rationally used; and 4) affordability: the extent to which a drug is reasonably priced and reimbursed. Scholarship and international organizations, such as the World Health Organization (WHO), have carefully deconstructed ATM from multiple angles. Horizontally, ATM is disentangled along slightly different dimensions. For instance, the WHO-MSH (2000, Center for Pharmaceutical Management 2003) uses a framework of availability, affordability, accessibility, and acceptability. Chaudhuri (2007) draws on the Indian case and proposes availability, affordability, and appropriateness as the three dimensions of ATM. Frost and Reich’s (2010) framework includes availability, affordability, and adoption. Vertically, as summarized by Bigdeli et al. (2013), constraints over ATM can be analyzed at five levels: 1) individuals, households, and community; 2) health service delivery; 3) health sector level; 4) public policies cutting across sectors; and 5) international and regional level. Hence, they advocate for a health system perspective to analyze complex and interconnected ATM barriers that span across different levels of a health system.

Despite these efforts, however, relevant studies on ATM usually adopt a normative, rather than historical, perspective. That is, ATM research mostly draws on public health theories and data to prescribe what infrastructures, resources, and measures *should* be put in place to improve ATM (e.g., Leach et al., 2005; Bigdeli et al., 2014). Little scholarship has addressed the fact that, depending on historical contexts, countries almost always prioritize certain dimensions of ATM over others in pharmaceutical governance. Even less attention is paid to exploring how and why this is the case. This article fills such gaps by not only deconstructing but also historicizing the concept of ATM. To do so, it focuses on drug availability and affordability, which are the two major dimensions of ATM that can pose trade-off problems for any state regulatory agenda. In particular, the author aims to make two arguments.

First, tensions easily arise between different dimensions of ATM, and prioritizing certain dimensions in pharmaceutical policy may impede improvements in others. For instance, there could be significant trade-offs between the goals of enhancing drug availability and improving drug affordability. Stressing affordability may result in aggressive price control mandates, which could discourage investment in new drug development or provision of low-priced essential medicines, thus potentially undermining drug availability in the long run. Second, which dimension(s) of ATM might be prioritized in the state’s pharmaceutical policy hinges upon social, economic, and political forces. The priority order changes along with shifts in these three historical conditions. For example, when a country is suffering from a serious drug cost inflation, it may prioritize affordability improvement over all else in pharmaceutical governance.

To substantiate these arguments, the author draws on interview and archival evidence from China. Specifically, the author gives a historical account of how and why pharmaceutical governance in China shifted its priorities regarding drug availability and affordability in the past decades. First, from 1949 to late 1970s, the Chinese government under Mao pursued both drug availability and affordability in its state-led reorganization of the pharmaceutical sector on the basis of a socialist planned economy. Second, from the early 1980s to 2015, China’s pharmaceutical policy first stressed drug availability by liberalizing the domestic industry in the early reform years but shifted toward prioritizing drug affordability over all else in the face of serious drug cost inflation after the mid-1990s. Third, from 2015 to the present has been the era of balancing innovation promotion and cost containment. Both availability and affordability have been stressed as the major priority by the central state, though coordination among different state agencies must be further improved.

By deconstructing and historizing the concept of ATM, the study not only highlights the tension between different facets of pharmaceutical access, but also reveals how such tension plays out in specific political, economic, and social contours. Although the Chinese case is more or less unique in that the state plays a heavy hand in almost all areas of social life, the pharmaceutical sector is also unique in that it is one of the most highly regulated sectors, to the extent that, even in the most laissez-faire countries such as the United States, the state’s role in pharmaceutical governance is indisputably strong. Hence, while the trade-offs between drug availability and affordability (and perhaps other dimensions of ATM) surely exist beyond China, they may take varied forms, stem from different historical roots, and provoke divergent state responses in other countries. Further research is needed to explore such variations. In any case, this paper takes a valuable first step toward unpacking how such trade-offs may play out in history.

## Theoretical Framework

A holistic and systematic view is certainly necessary for understanding ATM barriers synchronically. However, as social scientists, we need a framework that can set the research context for deconstructing ATM diachronically. In this paper, the author proposes a framework that helps divide ATM research into two research traditions with varying levels of analysis, different perspectives, and diverse methods. The first is the macroanalysis of the political economy of healthcare/pharmaceutical sectors, which conditions the two most commonly recognized pillars of ATM: drug availability and affordability. This research focuses on transnational or national laws, regulations, policies, and programs concerning drug development and manufacturing. In this line of work, scholars commonly used methods include historical and comparative analysis, interviews, and surveys. Researchers in this tradition have analyzed the political contests over the strength of drug patent regime (Parthasarathy, 2017; Shadlen, 2017), the challenges and opportunities faced by the development of neglected disease drugs (Moon et al., 2012; Craddock, 2017), the problematic knowledge production regime of drug discovery that disadvantaged populations in less developed countries (Pollock, 2019), the developmental foreign aid that helped low-income countries build drug production capabilities (Chorev, 2019), and the public insurance system’s battle against monopolistic drug prices set by Big Pharma (Scherer, 2004), just to name a few.

The second research tradition includes the meso- and microanalysis of the organizational, cultural, or community dynamics of healthcare/pharmaceutical delivery. Such dynamics shape the social constraints over drug accessibility and appropriateness (or other terms with similar connotations, such as acceptability, adaptability, and adoption). They include the rules and infrastructures for the distribution and reception of qualified drugs. Relevant research is usually conducted through methods such as interviews, surveys, ethnographic observation, and field experiment. Scholarship in this tradition has investigated the professional and civil movements for the expansion of access to medicines (Flynn, 2014; Harris, 2017), the suppressed local protest groups that could have joined transnational advocacy networks (Long, 2018), the racialized community health movements that advocated for alternative therapies (Decoteau, 2017 **)**, the incompetent procurement programs that failed to guarantee drug delivery for public hospitals (Chaudhuri, 2007), the health institutions and professionals that affected the prospect of drug rational use (Wirtz et al., 2013; Xiao et al., 2013), and so on. The framework proposed in this paper summarizes these two research traditions, as shown by Table 1 .

**TABLE 1:** Framework of ATM for social scientific research.

Social systems	Dimensions	Major relevant stages of drug life cycle	Levels of analysis	Analytical lenses	Methods
Social system of drug production	Availability	Drug development and manufacturing	Macro (global, national, regional)	Political economy of healthcare/ pharmaceutical sectors	Historical & comparative analysis, interview, survey, and statistics
Social system of drug distribution	Affordability	Drug pricing and reimbursement			
	Accessibility	Drug procurement and delivery	Meso and micro (community, household, individual)	Cultural, community, organizational dynamics of healthcare /pharmaceutical delivery	Interview, ethnography, survey, field experiment
	Appropriateness	Drug prescription and reception			

Building upon this framework, which has deconstructed ATM, the author takes one step further to historicize the concept. To do so, the author focuses on the tension between availability and affordability, the two primary goals of pharmaceutical governance at the macrolevel. In countries capable of drug production, governments often strive to develop an indigenous pharmaceutical industry. In particular, for Southern states whose firms mostly produce generic drugs, the aspiration for enhanced ATM is twofold. A booming local industry would significantly increase 1) the availability of drugs that have yet to be imported to the country and 2) the affordability of drugs that have been available but marketed at excessively high prices (often by Big Pharma). Here, this twofold ideal promises both availability and affordability. Yet, in practice, tensions easily arise between the two goals. For instance, aggressive price regulations may discourage investment in drug development or imports (Thomas, 2001). The author argues that, depending on the historical context, the state may prioritize one over another when designing and enforcing the rules of pharmaceutical governance. Several crucial questions are thus left unanswered: When and why would the state prioritize one dimension of ATM over others in its national pharmaceutical policy? Could the order of priority change over time? What are the driving forces of such change? To provide exploratory answers to these important questions, this paper examines ATM priority shifts between availability and affordability in China’s pharmaceutical governance from a macropoint of view. In the mid-20th century, China was one of the poorest countries in the world, but it has since grown into the world’s second largest pharmaceutical market with a huge, vibrant, local industry. It thus provides a convenient site for observing and analyzing historical changes in pharmaceutical governance priorities.

## Materials and Methods

The archival and interview data used in this paper were collected from a larger project exploring the political economy of the Chinese pharmaceutical sector between 2016 and 2018. First, archival data include law and policy documents, historical biographies, and media reports that reflect changes in pharmaceutical regulatory priorities. During the data collection for the larger research project, the author used a database called PKULaw^1^ and compiled 6,254 drug-related law and policy documents published by different Chinese state agencies and relevant media reports in the past four decades. For the purpose of this paper, the author selected all the 169 major documents concerning critical reforms issued by leading agencies in the sector for detailed analysis, such as those by the Ministry of Health (MOH), the drug administration, and the State Development Planning Commission (SDPC). The author also reviewed some historical biographies gathered from the author’s fieldwork in Beijing, such as the Report on the Development of the Pharmaceutical Industry in China: 1949–2009 published by the China Pharmaceutical Enterprise Management Association.

Second, the author drew the interview testimonies from 45 of the 156 interviews conducted for the larger project, which covered questions regarding the broader transformations in the political economy of the Chinese pharmaceutical sectors. The informants were recruited through purposive and snowball sampling, with a high response rate of 88.1% (156 out of 177 interview requests). The interviews were around 1.5 h long on average, and most of the interviews were recorded and transcribed. The author used the software Atlas.ti to store and analyze the transcripts and selected 45 interviews for this paper because they were the ones in which there were specifically asked questions about the priority shifts of drug regulation over time. These 45 interviews were collected in two groups of regions:

(1) 31 interviews from Beijing and Shanghai. In these regions, which are the political and economic centers of the country, the author interviewed former or current government officials, as well as policy consultants, who had witnessed crucial regulatory reforms in the past decades, and asked them to recall the primary goals of pharmaceutical reforms in certain periods and explain the reform dynamics.
(2) 14 interviews from selected cities and towns of Hebei, Jiangxi, Hubei, and Jiangsu Provinces. In these regions, which represented different levels of health institutions at the city, town, and rural levels, the author interviewed local health practitioners whose daily work had been impacted and asked them to describe their experiences with critical pharmaceutical reforms and shifting priorities with regard to ATM.

## Analysis

### China: Pharmaceutical Governance Changes in ATM Priorities

#### 1949—Late 1970s: Pursuing Drug Availability and Affordability Through Socialist Planning

The Chinese government under Mao pursued both drug availability and affordability in its state-led reorganization of the pharmaceutical sector on the basis of a socialist planned economy. Facing a serious drug shortage, the state tried to promote the local pharmaceutical development and production with full force to reduce import dependence. Meanwhile, like many other goods, local drugs were priced very low under central planning to satisfy the socialist ideal, and the medical reimbursement scheme achieved extensive coverage through state or collective insurance systems. Despite the persistent drug shortage, therefore, drugs were generally affordable at the time.

In 1949, when the Communist Party took power in China, the country was so devastated by long-lasting wars^2^ that it barely had an industrial base capable of producing Western medicines. For routine treatment, many people relied on traditional Chinese medicines. However, as poor public health and hygiene facilities exposed the world’s largest population to constant threats from deadly infectious diseases like *tuberculosis* and malaria, Chinese society desperately needed mass-produced antibiotics and other chemical drugs (Lee et al., 2009). One review of the terrible drug shortage described it as follows:

There were only 370 pharmaceutical factories nationwide, with merely 13,000 employees and pathetically meagre medical products except traditional medicines and simple gentian violet, merbromin (red potion), absorbent cotton, and gauze. Chemical drugs were in extreme shortage, which was exacerbated due to economic embargo by Western countries. The broad mass who just got emancipated faced a dire situation of “shortage of doctors and medicines” (queyi shaoyao). (China Pharmaceutical Enterprise Management Association, hereafter CPEMA, 2009, pp. 2, pp. 2)

Meanwhile, anticipating geopolitical conflicts and potential warfare in the near future, Mao’s government saw antibiotics as wartime strategic goods in an isolated economy. Hence, starting in the 1950s, the Mao government prioritized the development and production of active pharmaceutical ingredients (APIs) of chemical drugs, especially those of “antibiotics, sulfanilamide, febrifuge, vitamins, endemic medicines, and antituberculosis drugs” (CPEMA, 2009, pp. 3). Hence, the government incorporated antibiotics production into its centralized industrial plans. With Soviet assistance, it established Huabei Pharma (founded in Shijiazhuang, Hebei Province) as one of the 156 national industrial projects (Dong and Wu, 2004). Huabei Pharma was the largest state-owned pharmaceutical enterprise at the time. It was famed as one of the “Four Sons of the People’s Republic” along with Dongbei Pharma (Shenyang, Liaoning Province), Taiyuan Pharma (Taiyuan, Shanxi Province), and Xinhua Pharma (Jinan, Shandong Province).

In 1956, the central state reorganized the State Administration of Medicines (SAM) from the Ministry of Health (MOH) into the Ministry of Chemicals and Industry (MOCI), and the SAM began to take charge of pharmaceutical development, production, and distribution through central planning. Many of these efforts in new drug research and development were part of state-led military and diplomatic missions. Youyou Tu, China’s first Nobel prize winner in science, exemplified the scientific breakthrough of collective and socialist style. Tu and her team successfully synthesized artemisinin from traditional Chinese medicines, which was developed into the world’s most effective antimalaria drug and saved 200 million lives in the following decades (Tu, 2016). Additionally, there were other collective achievements, such as the creation of synthetic crystalline bovine insulin (Sun, 2015). Despite these occasional breakthroughs, however, “shortage of doctors and medicines” (*queyi shaoyao*) persisted as a salient problem due to a scarcity of capital, technology, and personnel in the Mao era. Research and production were practically paralyzed by the 10-year Cultural Revolution from 1966 to 1976, with intellectuals and professionals despised as antirevolutionary underdogs. Few factory leaders were educated enough to know anything about pharmaceutical development or manufacturing (Beijing, Interview 43).

On the distribution side, the socialist state played a heavy hand as well. Drugs were delivered to public health institutions through a three-tiered distribution system (regional, provincial, city) in fixed volumes, frequencies, and prices (Wei 2009). The Ministry of Health (MOH) was in charge of all of the publicly run health institutions (either nationalized or collectivized), which were responsible for prescribing and dispensing low-priced drugs to patients. During this period, with minimal professional and financial autonomy, public hospitals and doctors received fixed subsidies and salaries as civil servants working for the socialist government in the *danwei* system (Liu 2011). In principle, public hospitals could add a 15% markup to final drug prices to earn some profits (Sun et al., 2008), but since drug supplies were subject to strict central planning, this portion of revenue was also fixed.

In addition to the low and fixed prices, drug affordability was further guaranteed by the state or collective insurance schemes. Not long after the CCP took power, China established two urban schemes and one rural scheme to take responsibility for citizens’ medical expenses (Yu 2015): Government Insurance System (GIS, 1952) covering government employees, their dependents, and college students; Labor Insurance Scheme (LIS, 1951) for urban employers with 100 or more employees; Cooperative Medical Scheme (CMS, late 1950s) covering the majority of rural population on the basis of village communes. In the urban areas, people got their prescription drugs at hospital pharmacies with reimbursements from their employers (*danwei*). Hospitals would send the bills to their public employers, which covered the employees’ welfare costs from the womb to the tomb. The employees only need to pay for the registration fees ranging from 5 to 10 cents, although benefits can vary hugely across different types of enterprises (Beijing, Interview 34).

As for the CMS in the rural area, although it got praised as an unprecedented public health achievement by the WHO and other international observers, it was still far from adequate to address the unequal access to medicines between the urban and rural areas. With the serious drug shortage, most villagers could only access traditional medicines in the Mao era. A retired researcher affiliated with the MOH, who had rich experiences studying the CMS, explained that the collective commune leaders had the absolute authority to allocate the very limited medicine resources, “taking the good medicines (Western drugs), and leaving the mass herbal medicines” (Beijing, Interview 34).

Despite the widespread inequalities between different urban *danwei* communities and between urban and rural areas, however, it was almost never costly to seek medical service or drug prescriptions thanks to the very low, fixed prices and the extensive reimbursement system. Although the country was still suffering from serious drug shortage by the end of the 1970s, the state had been trying to improve both drug availability and affordability by exerting heavy intervention in each stage of drug supply, from development and production to distribution and reimbursement. It was true that the state only succeeded in achieving the latter aim due to the failure of isolated, planned economy, but its policies have stressed both goals. Such a balanced (though inefficient) policy regime would not change until the economic reform and opening began in the early 1980s.

#### Early 1980s to 2015: Priority Shifting From Drug Availability to Affordability

In the early reform years until the mid-1990, the priority of China’s pharmaceutical policy shifted toward enhancing drug availability following the trend of economic liberalization. Both central and local governments were motivated to not only privatize state-owned pharmaceutical enterprises gradually, but also establish joint-venture firms to attract foreign investment. The domestic industry quickly flourished as a result. However, the marketization of the healthcare and pharmaceutical sectors was rather uneven: while drug production became more and more privatized, drug distribution was still monopolized by public hospitals that remained under tight government command and control. As the state reduced subsidies for public hospitals and urged them to generate revenues from pharmacy business, such uneven reform led to dramatic drug cost inflation beginning around the mid-1990s. Worse still, the collapse of the old socialist insurance schemes took medical safety nets away from the majority of the population. Facing the rapidly growing medical costs, a large part of which was spent on pharmaceuticals, the discontented public pressured the state to step in. The government responded by pursuing new healthcare reforms in the following decades, which prioritized aggressive drug price control measures over all else in pharmaceutical policy design. As such, from the late 1990s to the early 2010s, enhancing drug affordability became the first and foremost political goal of China’s pharmaceutical governance, while availability was gradually sidelined in drug-related reforms.

To accommodate the reform and opening agenda, the Chinese state began to liberalize pharmaceutical production in the early 1980s. In this regard, the industry was no different than many other Chinese manufacturing sectors that embraced gradual, state-led liberalization. On the one hand, the State Administration of Medicines, the Drug Administration Department (DAD) under the Ministry of Health (MOH), and local governments all enforced developmental drug regulations to stimulate the extensive growth of local firms (Liu, 2011). For instance, the author’s interview data show that local drug approvals accelerated under loose quality standards. The agencies saw this as a strategy to kill two birds with one stone: boost the economy while increasing the drug supply. It did not take long for the local industry to significantly increase its productivity in manufacturing commonly used drugs, from vitamins to antibiotics, from APIs to generic formulations.

On the other hand, the government encouraged many Northern-based Big Pharma companies, such as Tianjin Otsuka, Shanghai Bristol-Myers-Squibb (BMS), Wuxi Huarui, Sino-US Tianjian Shike, Xi’an-Janssen, and Dalian Pfizer, to found joint ventures in China (China Pharmaceutical News, 2004). The central state also established China National Pharmaceutical Foreign Trade Corporation to facilitate foreign technology transfer and international cooperation. For example, with the assistance of the United Nations Development Program, Sichuan Antibiotics Institute and Beijing Institute of Pharmaceutical Preparations established research centers to nurture and screen antibiotics and other microbial drugs in 1986 (CPEMA, 2009). To better engage with the global market, China even yielded to United States pressure and installed a pro-MNC drug patent regime in 1992.

While the pharmaceutical reform in this period prioritized industrial prosperity, which contributed most to the improvement of drug availability among all the ATM dimensions, demand-side liberalization seriously lagged behind. The Chinese state began to retreat from the public healthcare sector at the beginning of the 1990s, reducing hospital subsidies to as low as 10% of their operating costs while maintaining strict price control over public medical services (Eggleston et al., 2008). Recognizing their financial pressure, the health ministry thus encouraged underfunded public hospitals and doctors to “generate revenues” from selling certain “advanced” drugs, medical examinations, and specialist services that could be priced high at discretion. Such partial price liberalization forced underfunded public hospitals to exploit their monopoly over pharmacies, seeking drug profits and commercial rebates from pharmaceutical firms as a major revenue source.

In addition to this new medical financing model “feeding the hospitals with drug sales” (*yiyao yangyi*), another factor also pushed up drug expenses: following import liberalization in the 1980s, Big Pharma often marketed their monopolistic import drugs at a very high price. For instance, Roche’s famous brand-name drug Rocephine (ceftriaxone) could be several dozen or even a hundred times more expensive than its local generics (Jiangxi, Interview 9; Beijing, Interview 38). Not surprisingly, total health expenditures grew fast after the mid-1990s, with an average of 45.7% spent on pharmaceuticals between 1990 and 2009 (Shi et al., 2014).

To make matters worse, the old socialist welfare regime collapsed around the same time. The LIS that used to cover medical costs of urban employees working in the *danwei* system began to fall apart as many debt-ridden SOEs underwent market reform and reorganization in the 1990s. Hundreds of millions of SOE workers lost their “iron bowls” and the accompanying benefits in just a few years, finding no way to get their medical bills reimbursed. As for the rural area, the old CMS collapsed even earlier in the 1980s, when people’s commune and collective farming came to the end. Indeed, thanks to the economic reform, township and village pharmaceutical enterprises, drugstores, and rural clinics boomed and greatly enhanced the availability of basic Western medicines in villages. However, farmers also began to feel the burden of rapidly increasing out-of-pocket drug costs after the mid-1990s, whether they remained in the village or migrated to the city for new jobs. Their financial burden was only stronger than urban counterparts due to the depreciation of their agricultural products and the decline of township and village enterprises (Beijing, Interview 34).

Starting around the mid-1990s, therefore, the biggest headache for pharmaceutical governance became fast-growing drug costs. Public outrage pressured the state to react. While open protests were rare, media reports about rising expenses and public complaints attracted the attention of the central state (Beijing, Interview 38). To appease the public, a few state ministries moved to address the problem. There were three major price control measures in this period. One was the drug retail price regulations, enforced by the State Development Planning Commission (SDPC, reorganized into National Development and Reform Commission—hereafter NDRC—in 2003). The other two were centralized drug bidding platforms and National Essential Medicine System (NEMS), both launched by the Ministry of Health (MOH). As the two agencies leading healthcare reforms, the SDPC and the MOH were able to set aggressive price control as the major goal of pharmaceutical governance.

The SDPC’s move began in 1996. To tackle the “market price disorder” in the pharmaceutical sector and appease a discontented public, the central state resumed its price-setting power and assigned this power to the Pharmaceutical Office of the Price Department at the SDPC. The Office issued a catalog covering drugs “of large amount and wide application,” which included some newly imported and commonly used drugs in the market (Dong and Wu, 2011, pp. 31). Drug prices at different stages of circulation, including factory price, procurement price, and retail price, were all set by the catalog. Although the SDPC relinquished this price-setting power in 2000, it mandated that retail prices for over 2000 drugs be reduced by 15–20% more than 20 times between 1996 and 2007 (Dong and Wu, 2011). Even if these measures turned out to have had a very limited impact on inflated drug expenditures, they represented the most prominent pricing regulations enforced by the SDPC.

Additionally, the SDPC (reorganized into NDRC in 2003) retained its power to cap drug retail prices after 2000. It stipulated that drugs with specific therapeutic or economic values could apply for the status of “separate pricing,” which would allow for a higher retail price ceiling.^3^ However, such rules could be rather arbitrary, with the NDRC officials enjoying huge pricing discretion. Despite being justified as a cost-saving measure, this practice only nurtured a hotbed for rent seeking. By 2015, all of the five major officials in the Office had been arrested and accused of corruption.^4^ Only then did the NDRC forfeit its price-setting power, which was criticized by my interviewees as unnecessary, corruptive, and cost-inducing:

For example, there was a “differential pricing” principle. What was a differential pricing principle? Let’s say, tablets, capsules, granules, and sustained release agents were all different dosage forms of an active ingredient. How to price them? It sets price for one piece, for example, one ordinary piece (e.g., tablet) and then sets other forms’ prices based on a coefficient. Very complicated. People couldn’t understand what he had done. Even the Prime Minister did not understand! … Too complicated, too much detail! Very technical. In the end, only he (the rule maker) can understand. Many dirty tricks in it! (Beijing, Interview 45)

Although such policies consistently proved to be ineffective and even counterproductive as the cost of corruption ultimately reflected in the final price, aggressive price control remained an orthodoxy for central state agencies. Among them was the most powerful MOH. Instead of loosening price control over medical services to grant more financial autonomy to public hospitals so that they might reduce dependence on drug revenues, the MOH chose to reinforce its command and control over hospital pharmacy business. It adopted two aggressive drug price control measures.

First, it experimented with centralized drug bidding and procurement platforms (referred to as centralized bidding platforms hereafter) to crack down on inflated drug prices and commercial rebates. Throughout the 2000s, the MOH kept promulgating documents^5^ in the hope of perfecting rules for such platforms. Moreover, after the central government launched the new healthcare reform plan in 2009,^6^ the MOH further centralized the bidding platforms at the provincial level.^7^ The procurement principle of “low prices trump all” (*wei dijia shiqu*), which originated with the Anhui Province’s “two-envelope” system, gained wide popularity in many provinces. This principle allowed government bidding platforms to put an excessive emphasis on price over quality, thus often leading to a race to the bottom among local firms (Mossialos et al., 2016).

Second, the MOH established a National Essential Medicine System (NEMS), which was one of the top five priorities in the 2009 healthcare reform plan. In this new system, the logic of prioritizing price cuts over all else became even more salient. The MOH created a national essential medicines list that covered 307 drugs, and it required all of China’s primary care institutions to implement the NEMS and sell drugs at zero markup within three years (Guan et al., 2011). Although many of the essential drugs were already very cheap before the reform, the producers had to engage in cut-throat price wars on the bidding platforms. As such, while these medicines’ procurement and retail prices decreased, problems such as substandard quality and drug shortage became very common at the grassroots level (Liu et al., 2017; Beijing, Interview 16; Hebei, Interview 25). Echoing some quantitative findings (Fang et al., 2013; Liu et al., 2017), the author’s fieldwork revealed that drug availability plummeted in township and rural clinics. In the past, the majority of these primary care institutions’ revenues came from drug sales, with the drug markup averaging 40.5% (Guan et al., 2011). With few advanced technologies or specialists, they mainly treated common diseases by prescribing drugs. Under the NEMS mandates and zero markup policy, however, many of these institutions fell into significant financial trouble, since their major revenue source was cut off without sufficient and timely government subsidies to make up the loss. As a township clinic head complained:

Patients would come and ask for certain drugs, those with chronic diseases like high blood pressure or diabetes. Or just cold and stomachache. But you can see us here. Look, this is our drug shelf. All drugs (are) here. We don’t even have enough drugs for stomachache! Even their rural clinics have four kinds of stomach drugs! How many do we have? One! Only one! Nonsense! ... My salary is so low here. I would not let my son be a doctor. Never! Our operation is very difficult here. Just some basic public health service, but how are we gonna feed ourselves? Before, we can cut the procurement price and save the markups for ourselves. The price was not high at all! We all know the market price. (Hebei, Interview 25)

In sum, even though other dimensions of ATM were mentioned in formal policies, the priority of actual pharmaceutical governance shifted from availability to affordability after the mid-1990s. While the three price control measures—price caps, centralized drug bidding platforms, and NEMS—seem to have reduced the nominal prices of many generic drugs, they failed to contain the inflation of either drug costs or overall health expenditures (Liu et al., 2017). More importantly, they often unexpectedly undermined drug availability at the grassroots level. Notably, few price control measures were targeted against Big Pharma’s brand-name drugs, which enjoyed “supranational treatment” by claiming superior technology/quality over generic products (Mossialos et al., 2016; Beijing, Interview 40).

#### 2015—Present: Rebalancing Availability and Affordability

Beginning in 2015, however, China’s pharmaceutical governance entered the era of balancing innovation promotion and cost containment. A series of drug regulatory reforms set drug availability as a top priority along with drug affordability. This time, the availability of cutting-edge medicines to treat deadly diseases such as cancer, rather than that of commonly used drugs like antibiotics, was at stake. It is the first time since the economic reform that the Chinese government began to strive for a balance between drug availability and affordability. What social, economic, and political forces account for this progress? On one hand, as cancer became a leading cause of death in China, the public demand for cutting-edge medicines grew dramatically. On the other hand, the national industrial policy aimed at transforming the economy from a manufacturing giant to an innovative powerhouse, and in the pharmaceutical sector, it encouraged local firms to shift investment from generic production to new drug development. In response, the state assigned two agencies to accommodate these socioeconomic demands: it empowered the central drug administration to improve new drug availability through registration reforms, and it established a new State Medical Insurance Administration (SMIA) as the most powerful agency in drug pricing regulations in replace of the MOH. One of SMIA’s major goals was to increase drug affordability without discouraging new drugs from entering the market.

First, the increasing demand for cutting-edge cancer drugs gained public prominence in late 2014 during a national sensation: the criminal charge against Lu Yong, the “first broker of Indian cancer drugs” (Hong, 2015). As a leukemia patient, Lu has survived on the Indian generic Gleevec since 2004 (for more details, see Yang, 2014). Out of altruism, he also helped thousands of fellow patients obtain Indian Gleevec (unlicensed in China, labeled as fake) via informal brokerage. After getting caught, he was accused of the crime of selling fake drugs. The media exposure of this criminal charge turned his case into a national sensation overnight, and the procuratorate eventually dropped the charge under great public pressure (Hong, 2015). For the first time, the Chinese public came to realize that there had been a huge gray market of transnational cancer drug brokerage and that it was the only life-saving channel for many desperate patients. Lu’s case not only revealed the striking price discrepancy between Chinese and Indian cancer drugs, but also exposed the lengthy drug lags that had inhibited the availability of innovative medicines for years.

Before 2015, it took a new drug 5 years on average to gain drug approvals from the day the application was submitted.^8^ Cutting-edge drugs developed by Big Pharma, which had gained approval in Northern markets, were forced to redo clinical trials in China to be eligible for market entry. Chinese people suddenly learned that foreign drug providers like those in India not only sold drugs of much cheaper prices, but also offered the latest therapies, which had not been available in the Chinese market (Beijing, Interview 2; Jiangsu, Interview 29). Since the exposure of Lu’s case, the inadequate availability and affordability of life-saving drugs have gained increasing media and public attention. In summer 2018, the release of the top-rated blockbuster movie *Dying to Survive* (*wo bushi yaoshen*) further amplified the call for reform. Adapted from Lu’s case, the movie dramatized the struggles of informal drug brokers and dying cancer patients. Becoming one of the best-selling movies in Chinese history, it provoked widespread public discussion over the role of the state in satisfying unmet local medical needs. One of the major debates became about what government action is required to achieve a balance between drug availability (innovation promotion) and affordability (generic drug provision).^9^

Besides changing disease burden and societal demand, macroeconomic policy was another driving force of the priority shift. Around 2015, President Xi pushed forward economic plans such as “Supply side Reform” (*gongji ce gaige*) and “Made in China 2025.” For higher value-added products, such as electronic chips and medical devices, China had been heavily reliant on foreign imports. The core ideal of these reforms was to reduce such reliance and steer Chinese industries toward innovation-oriented transformation. Local firms would be further propelled to compete with international giants on advanced products. The pharmaceutical industry was listed as one of the strategic sectors for this ambition. To accommodate the reform agenda, the government increased funding for innovation promotion programs such as the National Science and Technology Special Project for “New Drug Development,”^10^ launched by the Ministry of Science and Technology (MOST) as part of the 12th five-year plan.

In pursuit of the new policy goals, the State Council assigned Bi Jingquan as the leader of the China Food and Drug Administration (CFDA) in 2015. Bi has served as the deputy secretary of the State Council and the deputy director of the National Development and Reform Commission (NDRC). Thanks to his political clout, Bi soon moved to echo the macroeconomic policy in the arena of drug regulation, as well as addressing the public’s growing medicine demand (Beijing, Interview 37). After Bi took office, the empowered CFDA identified the lengthy drug lag as a major obstacle to improving new drug availability in China. As explained by a CFDA official:

At that time, the focus was on how to allow common people to use innovative and good medicines as soon as possible. This was a consistent idea. Like our center’s research work (aiming) to protect and promote public health. Our mission was to make good medicines and drugs available in the global market enter the Chinese market more quickly. Why was it proposed as such? Because before, indeed, some provisions in the Registration Measures and the Drug Administration Law were not conducive to the accelerated market entry of foreign drugs in China. Some of the procedural settings were not in line with the international standards. (Beijing, Interview 37)

To improve drug availability, the agency launched aggressive reforms to streamline the registration procedures, encourage pharmaceutical innovation, and accelerate review speed.^11^ For instance, it joined the International Council for Harmonisation of Technical Requirements for Pharmaceuticals for Human Use (ICH) in 2017. In doing so, it made the Chinese drug registration regime much more compliant with international conventions (e.g., recognizing clinical data collected overseas as evidence for drug safety and efficacy). The agency also simplified the registration procedures by removing the requirement of administrative approvals for conducting clinical trials.^12^ In a word, the CFDA prioritized improving the availability of innovative drugs in their groundbreaking reforms.

Yet the updated priority did not come at the expense of loosening drug cost control. Another crucial regulatory change took place on the demand side. The new SMIA, established in 2018, took over all authority on drug procurement from the MOH and the Ministry of Human Resources and Social Security (MOHRSS). Tasked with containing the inflation of drug costs while encouraging the market entry of cutting-edge therapies, the SMIA became the most powerful agency in the sector. In 2018, while commenting on the movie *Dying to Survive*, Prime Minister Li Keqiang promised to do more in addition to removing tariffs over import cancer drugs.^13^ Soon, the SMIA, which was endowed with strong institutional purchasing power, seized the political opportunity to push forward negotiations with pharmaceutical firms (Beijing, Interview 40). It added 17 cutting-edge cancer drugs to the national reimbursement list, with an average price cut of 56.4%.^14^ Moreover, the SMIA began to experiment with national drug bulk-buy schemes (*dailiang caigou*) in major localities around the same time, which aimed to negotiate unprecedented price cuts with both MNCs and domestic drug producers upon the promise of extensive insurance coverage. Along with the CFDA’s reform, such moves exemplified the Chinese state’s radical efforts to strike for a rebalance between availability and affordability in the rules of pharmaceutical governance. Both foreign and local firms were incentivized to speed up the development and registration of innovative drugs, which would earn large market shares once covered by the SMIA’s insurance reimbursement list.

## Discussion

The author has shown how Chinese pharmaceutical governance priorities shifted between drug availability and affordability over time and finds that, in the Mao era, the socialist regime prioritized both drug availability and affordability in the central planning of the pharmaceutical sector. With the launch of the reform and opening in the 1980s, the Chinese state began to put more emphasis on availability through the liberalization of pharmaceutical production. However, the state gradually shifted the priority to affordability after the mid-1990s, when drug price and cost inflations became a major financial burden on patients. Only after 2015 did the state launch another round of pharmaceutical reforms to strive for a rebalance between drug availability and affordability.

This case substantiates the importance of deconstructing and historicizing the ATM: enhancing ATM can be a task that poses very different expectations for different governments across historical periods. By analyzing the Chinese case, the author shows that shifts in social, economic, and political forces collectively contribute to changes in the state’s perception of what is the most urgent priority. A major limitation of this study is that it only involves two dimensions of ATM, whereas the incorporation of the other two dimensions (drug accessibility and appropriateness) may further enrich and complicate the analysis. Also, looking forward, the author calls for additional research on whether and to what extent this framework can apply to other contexts. We need more inquiries into how and why states may change the priority of pharmaceutical governance with regard to ATM over time. Only through such historical analysis can we have a better understanding of the opportunities and challenges presented for people aspiring for better ATM in the present and future era.

## Data Availability Statement

The datasets generated for this study will not be made publicly available. The interview data are protected for confidentiality.

## Author Contributions

The author confirms being the sole contributor of this work and has approved it for publication.

## Conflict of Interest

The author declares that the research was conducted in the absence of any commercial or financial relationships that could be construed as a potential conflict of interest.

## Footnotes

1 PKULaw is a bilingual database for searching China’s laws and regulations, cases, journal articles, and gazettes. It has been widely used as a credible database for socio-legal research in the Chinese context. The author accessed this database through the Peking University Library. See http://www.pkulaw.cn/

2 The most recent ones included the Japanese invasion from 1937 to 1945 during World War II and the civil war between the Nationalist Party and the Communist Party of China from 1945 to 1949.

3 Notification of the SDPC on Questions about Separate Drug Pricing (No. 13), on January 4, 2001.

4 See http://m.y-lp.com/pages/Article.aspx?id=5118500835304745641

5 A few milestone documents include the following: Notification of Working Norms on Pharmaceutical Bidding and Procurement Process in Medical Institutions (Interim Procedure) (No. 308) on November 12, 2001 and Notification of Some Regulation on Further Standardization of Pharmaceutical Bidding System and Procurement Process (No. 320) on September 23, 2004.

6 Opinions of the CPC Central Committee and the State Council on Deepening the Health Care System Reform (No. 6) on March 17, 2009.

7 Working Norms on Pharmaceutical Bidding and Procurement Process in Medical Institutions (No. 64) on July 7, 2010. Guiding Opinions on Establishing and Standardizing Essential Drug Procurement Process in Public Primary Healthcare Institutions (No. 56) on November 19, 2010.

8 For more detail, see the report by Insight, http://yao.dxy.cn/article/92630?trace=related

9 For instance, a law firm cited the movie in the beginning of its blueprint for the reform of China’s pharmaceutical sector. See https://www.cliffordchance.com/content/dam/cliffordchance/briefings/2019/11/chinas-blueprint-for-its-pharmaceutical-sector-chinese-version.pdf

10
http://www.nmp.gov.cn/zxjs/zdxy/201012/t20101208_2128.htm

11 See the landmark Opinions of the State Council on Reform of the System of Evaluation, Review and Approval of Drugs and Medical Devices (No. 44), on August 9, 2015.

12 The reform formally came into effect on July 27, 2018. See http://www.nmpa.gov.cn/WS04/CL2111/329716.html

13 See http://www.gov.cn/guowuyuan/2018-04/13/content_5282287.htm

14 See http://society.people.com.cn/n1/2018/1011/c1008-30333639.html
